# L‐Shaped Association Between Composite Dietary Antioxidant Index and Risk of Dementia: Results from a Prospective Cohort Study

**DOI:** 10.1002/alz70860_099306

**Published:** 2025-12-23

**Authors:** Caimei Luo, Ruihan Wang, Feng Yang, Linyuan Qin, Hui Gao, Qin Chen

**Affiliations:** ^1^ West China Hospital of Sichuan University, Chengdu, Sichuan, China

## Abstract

**Background:**

Composite dietary antioxidant index (CDAI) is a critical metric for assessing antioxidant‐rich diets. However, the relationship between the CDAI and dementia has not yet been explored. This study aimed to investigate the association between CDAI and the risk of dementia and dementia subtypes, as well as the underlying mechanisms involved.

**Methods:**

A total of 157,742 dementia‐free participants from the UK Biobank were prospectively analyzed. CDAI was calculated based on the intake of six dietary antioxidants derived from dietary information using a 24‐hour recall questionnaire. Cox proportional hazards models were used to investigate the associations between CDAI and the risk of all‐cause dementia, Alzheimer's disease (AD), and vascular dementia (VD). Restricted cubic splines were used to examine potential non‐linear correlations. Multivariable linear regression and mediation analyses were conducted to explore the underlying mechanisms, focusing on blood inflammation markers and brain structures.

**Results:**

During a median follow‐up of 13.39 years, 1,822 participants (1.16%) were diagnosed with dementia, including 791 cases of AD and 322 cases of VD. After multivariable adjustment, higher CDAI scores were significantly associated with a decreased risk of all‐cause dementia and AD. An L‐shaped nonlinear association was found between CDAI scores and both all‐cause dementia and AD, with inflection points at 1.579 for all‐cause dementia and 0.848 for AD. Below the inflection points, each unit increase in CDAI was associated with a 6.3% reduced risk of all‐cause dementia (HR, 0.937; 95% CI: 0.907‐0.968, *p* < 0.001) and a 6.5% reduced risk of AD (HR, 0.935; 95% CI: 0.897‐0.974, *p* = 0.001). Blood inflammation markers partially mediate the relationship between CDAI and all‐cause dementia and AD. CDAI was positively associated with cortical and subcortical volumes in the lower CDAI group after Bonferroni correction. No mediating effects of the brain volume were observed.

**Conclusions:**

There was an L‐shaped nonlinear association between CDAI scores and the risk of all‐cause dementia and AD. The CDAI has potential value as a biomarker for dementia and a dietary intervention target for reducing dementia risk. The beneficial effects of CDAI on inflammatory markers and brain structure provide valuable insights into the underlying mechanisms. However, further studies are required to confirm these results.

